# Is Adipose Tissue a Place for *Mycobacterium tuberculosis* Persistence?

**DOI:** 10.1371/journal.pone.0000043

**Published:** 2006-12-20

**Authors:** Olivier Neyrolles, Rogelio Hernández-Pando, France Pietri-Rouxel, Paul Fornès, Ludovic Tailleux, Jorge Alberto Barrios Payán, Elisabeth Pivert, Yann Bordat, Diane Aguilar, Marie-Christine Prévost, Caroline Petit, Brigitte Gicquel

**Affiliations:** 1 Genetics and Biochemistry of Microorganisms, Centre National de la Recherche Scientifique (CNRS) Paris, France; 2 Unit of Mycobacterial Genetics, Institut Pasteur Paris, France; 3 Department of Pathology, Instituto Nacional de Ciencias Medicas y Nutricion Tlalpan, Mexico; 4 Department of Infectious Diseases, Institut Cochin Paris, France; 5 Hôpital Européen Georges-Pompidou, Assistance Publique-Hôpitaux de Paris, Department of Anatomo-Pathology, Paris, France; 6 Laboratory of Electron Microscopy, Institut Pasteur Paris, France; University of Washington, United States of America

## Abstract

**Background:**

*Mycobacterium tuberculosis*, the etiological agent of tuberculosis (TB), has the ability to persist in its human host for exceptionally long periods of time. However, little is known about the location of the bacilli in latently infected individuals. Long-term mycobacterial persistence in the lungs has been reported, but this may not sufficiently account for strictly extra-pulmonary TB, which represents 10–15% of the reactivation cases.

**Methodology/Principal Findings:**

We applied *in situ* and conventional PCR to sections of adipose tissue samples of various anatomical origins from 19 individuals from Mexico and 20 from France who had died from causes other than TB. *M. tuberculosis* DNA could be detected by either or both techniques in fat tissue surrounding the kidneys, the stomach, the lymph nodes, the heart and the skin in 9/57 Mexican samples (6/19 individuals), and in 8/26 French samples (6/20 individuals). In addition, mycobacteria could be immuno-detected in perinodal adipose tissue of 1 out of 3 biopsy samples from individuals with active TB. *In vitro*, using a combination of adipose cell models, including the widely used murine adipose cell line 3T3-L1, as well as primary human adipocytes, we show that after binding to scavenger receptors, *M. tuberculosis* can enter within adipocytes, where it accumulates intracytoplasmic lipid inclusions and survives in a non-replicating state that is insensitive to the major anti-mycobacterial drug isoniazid.

**Conclusions/Significance:**

Given the abundance and the wide distribution of the adipose tissue throughout the body, our results suggest that this tissue, among others, might constitute a vast reservoir where the tubercle bacillus could persist for long periods of time, and avoid both killing by antimicrobials and recognition by the host immune system. In addition, *M. tuberculosis*-infected adipocytes might provide a new model to investigate dormancy and to evaluate new drugs for the treatment of persistent infection.

## Introduction

Up to one third of the world's population is estimated to carry latent *M. tuberculosis* infections, and hundreds of millions of tuberculosis (TB) reactivation cases are anticipated in the coming decades if control is not strengthened [1], [2]. This is particularly true in areas of low or moderate endemicity, where most cases of active TB result from reactivation of a latent infections [3], [4].

One important and historical question regarding TB latency deals with the location of the bacilli during that period of the disease [5]. Numerous authors addressed this issue in the early part of the past century using lung and lymph node necropsy samples from individuals who had died of causes other than TB [6]. As a proof of the high incidence of TB in Europe at the time, tuberculous lesions were observed in most samples. Depending on the studies, variable percentages of such lesions were found to contain viable and infectious bacilli, as assessed by *in vitro* culture or inoculation to guinea pigs [6]. Of particular interest is a study by Opie and Aronson (1927) who reported that in addition to old lesions, unaffected portions of the lungs may also host persistent bacilli [7]. These results have been recently confirmed and expanded by Hernández-Pando *et al*. (2000), who reported *in situ* PCR-detection of *M. tuberculosis* DNA in normal-appearing lung tissues from about 35% of the necropsy specimen included in the study [8].

Apart from the lungs and the lymph nodes, other organs and tissues are likely to host persistent bacilli during TB latency. Indeed, a striking feature of reactivating TB is that nearly 15% of the cases occur at extra-pulmonary sites, including the brain, the meninges and the spinal cord, the skin, the internal organs, and the genitourinary tract, without apparent pathology in the lungs [9], [10]. In those cases, it is most likely that growth of the bacilli resumes directly from the reactivation foci rather than from pulmonary sites and subsequent migration of the bacteria to other body sites.

The adipose tissue constitutes 15–25% of the total body mass and is broadly distributed throughout the body. Possible interactions between this tissue and mycobacteria have not been rigorously investigated. Here, we have investigated these interactions by using a combination of adipose tissue models, including the widely used 3T3-L1 murine adipose cell line as well as human primary adipocytes and adipose tissues from patients with active pulmonary TB or from individuals who died of causes other than TB. Altogether, our results suggest that the adipose tissue might constitute one important mycobacterial reservoir in which the tubercle bacillus could persist in a dormancy-like state and avoid both anti-mycobacterial drugs and host defence mechanisms.

## Methods

### Bacteria and cells

*M. tuberculosis* strains were propagated at 37°C in complete Middlebrook 7H9 medium. 3T3-L1 fibroblasts were cultured in DMEM (Invitrogen) containing 10% heat-inactivated foetal calf serum (FCS; Dutscher), at 37°C under 5% CO_2_. At confluency, pre-adipocytes stop dividing. Adipocyte differentiation was initiated at confluency (day 0) by adding 100 mM 3-isobutyl-1-methylxanthine (IBMX), 250 nM dexamethasone (DM), and 1 µg/ml bovine insulin (all from Sigma) into the culture medium. At day 2, medium was replaced by DMEM-10% FCS containing 1 µg/ml insulin only. Full differentiation was reached at day 8–10, as assessed by red oil staining of lipid droplets. Human adipose cells obtained from plastic surgery wastes were differentiated *in vitro* using appropriate differentiation and nutrition media (PromoCell).

### Mycobacterial intracellular survival and binding experiments

Cells were infected at a multiplicity of infection (MOI) of 1 bacterium per cell for 4 h at 37°C, washed with serum-free DMEM, and further incubated with fresh complete medium. Mature adipocytes were infected at day 10 after differentiation. At various time-points, cells were washed with serum-free DMEM, lysed in distilled water containing 0.01% Triton X-100 (Sigma), and the lysates were plated at various dilutions onto 7H11 agar. In some instances, cells were incubated with 200 µg/ml amikacin 2 h prior lysis in order to kill extra-cellular bacteria. Plates were incubated at 37°C for 18–20 days after which colony-forming units (CFUs) were scored. For binding experiments, cells that had been pre-incubated with various inhibitors for 30 min at 4°C, were infected with bacteria at a MOI of 1 for 4 h at 4°C in the presence of the inhibitors, washed in serum-free DMEM, and treated as described above for CFUs scoring. Fucoidan, polyadenosinic acid, and polyinosinic acid (all from Sigma) were used at 0.1 mg/ml. PAz-PC (Cayman), a major component of oxidized low-density lipoprotein was from used at 0.01 mg/ml. All antibiotics used in the study were from Sigma. For quantification of mycobacterial chromosome equivalents (CEQs) in infected cells, we followed a previously described procedure for quantitative real-time PCR amplification of the *sigF* gene [11].

### Electron microscopy

Cells were fixed in 2.5% glutaraldehyde in 0.1 M cacodylate buffer, pH 7.4, for one hour at room temperature. After 3 washes in the same buffer, cells were postfixed in 1% osmium tetroxide in 0.1 M cacodylate buffer, pH 7.2, for 1 hour at room temperature. The cells were then washed three times in cacodylate buffer, rinsed in 30% methanol, and stained for 1 hour in uranyl acetate. The cells were then dehydrated in a graded series of ethanol solutions (from 25 to 100%) before being embedded in Spurr resin at 60°C. Ultrathin sections were cut on an Ultracut UCT microtome (Leica) and then examined under a JEM1010 microscope (Jeol) at 80 Kv accelarating voltage. For immuno-staining for perilipin A/B, the cells were fixed in 3% paraformaldehyde and 0.2% glutaraldehyde in 0.1 M phosphate-buffered saline (PBS), pH 7.4, for 1 hour at room temperature. The cells were washed three times in the same buffer before embedded in 10% gelatine and then infused in 1.7 M sucrose +15% polyvinyl pyrolidone for 4 hours at 4°C. Cells were then cryosectionned in Ultracut FCS microtome (Leica) at −130°C, and then immunolabelled, after quenching of free aldehyde groups and blocking in 0.1% BSA in PBS. Cryosections were incubated in anti-perilipinA/B rabbit IgGs (Affinity BioReagents) used at 1/100 dilution in 0.1% BSA in PBS containing 0.1% fish gelatin (PBG), for 1 hour at room temperature. After six washes in PBS containing acetylated BSA (Aurion), cells were incubated with a goat-anti-rabbit F(ab′)2 fragments conjugated to 10 nm gold particles (BBinternational) used at 1/25 dilution in PBG. The preparations were negatively stained with uranyl acetate, embedded in methyl cellulose following the Tokuyasu method, and observed under the microscope.

### Human samples

Individuals who had died from causes other than tuberculosis were autopsied in the Department of Pathology at the General Hospital of Mexico, or in the Department of Anatomo-Pathology at the Georges Pompidou European Hospital of Paris. As soon as possible after death, blocks of various tissues from 3 to 5 cm were sectioned and fixed in 10% formaldehyde dissolved in PBS. After fixation, the tissues were dehydrated in graded ethyl alcohol concentrations and embedded in paraffin. For histological examination, 5 µm sections stained with hematoxylin and eosin were obtained. The same paraffin blocks were used for mycobacterial DNA localization by *in situ*-PCR. Tissues were obtained for the exclusive purpose of the legally authorized autopsy and no additional samples were taken for the purpose of the present study. Lymph nodes from three patients with active TB were referred to the Laboratory of Pathology at the Saint-Louis Hospital (Paris, France) for the purpose of TB diagnosis with the patient's consent and used according to institutional guidelines. Treatment of the biopsy and immunodetection of *M. tuberculosis* antigens were achieved as previously published [12].

### *In situ* and conventional PCR

Five microns width sections from the paraffin blocks were mounted on silane coated slides, deparaffinized for 18 h at 60°C and sequentially immersed in xylene (30 min at 37°C), absolute ethanol, 75%, 50%, 25% ethanol and water. Cells were rendered permeable by incubation for 10 min at room temperature in 0.02 M HCl. Then proteins were depleted by incubation with proteinase K 1 µg/ml (Gibco) for 30 min at 37°C. The proteinase was then inactivated by boiling in a microwave for 15 sec and the section plunged immediately into 20% acetic acid for 15 sec to inactivate endogenous alkaline phosphatase. The polymerase chain reaction was carried out by incubating the sections with 50 µl of 1× reaction buffer (Gibco, BRL), 1.5 U Taq polymerase, 2 µM MgCl_2_, 40 µM dNTP, and 0.2 µM dUTP labeled with digoxigenin (Boehringer Manheim), and 60 pg each of IS6110 *M. tuberculosis* insertion sequence primers. The sequence of the primers was: 5′-CCCTGCGAGCGTAGGCGTCGG-3′sense and 5′-CTCGTCCAGCGCCGCTTCGG-3′antisense. The slides were sealed using the Assembly tool (Perkin Elmer) and placed in the thermocycler (Touch Down, Hybaid). The *M. tuberculosis* DNA amplification started with denaturation at 94°C for 1 min, annealing at 70°C for 1 min, and extension at 72°C for 1 min, for 35 cycles. PCR products were detected with sheep anti-digoxigenin antibodies coupled to alkaline phosphatase (Boehringer Manheim) diluted 1/500. The chromogen was 5 bromo-4-chloro-3-3 indolyl phosphate toluidine salt (BCIP) and tetrazolium nitroblue (Boehringer Manheim) diluted 1/50. Sections were counterstained with nuclear fast red to avoid any interference with the blue signal generated by mycobacterial DNA in the *in situ* PCR. Lung sections from 6 tuberculous autopsies were used as positive controls. The negative control consisted of performing the whole procedure with non-infected mouse lung. A further control was the use, on the same slide as the test section, of a duplicate section to which PCR mix was added without Taq and another section added without primers. Conventional PCR to amplify IS*6110* in tissue extracts was performed as previously described [8].

## Results

### *M. tuberculosis* binds to adipocytes through scavenger receptors

**Figure 1:**
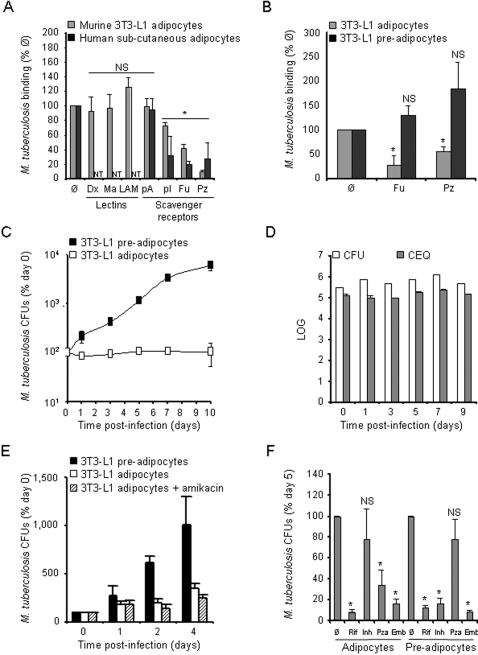
*M. tuberculosis* persists in a non-replicating state inside adipocytes. **A.** 3T3-L1 mouse (grey bars) and primary human (black bars) adipocytes were infected with *M. tuberculosis*, clinical isolate MT103, at a MOI of 1. Mycobacterial binding to the cells was measured after 4 h at 4°C in the presence of various inhibitors. Man, yeast mannan; LAM, lipoarabinomannan; pA, polyadenosinic acid; pI, polyinosinic acid; Fuc, fucoidan; PC, PAz-PC, a predominant form of oxidized low-density lipoprotein. Results are expressed as % of the binding without inhibitor (Ø). **B.** 3T3-L1 adipocytes (grey bars) and pre-adipocytes (black bars) were infected as described in A. in the presence of Fuc or PAz-PC. Mycobacterial binding was assessed and analysed as in A. **C.** 3T3-L1 pre-adipocytes (black squares) and adipocytes (open squares) were pulsed with *M. tuberculosis* MT103 at a MOI of 1 for 4 h at 37°C, then chased with fresh medium. Mycobacterial survival, expressed as % of the bacterial load at day 0, was monitored on a 10-day period. The results are standardized as % of the bacterial load at day 0 because of the differential *M. tuberculosis* binding to adipocytes and pre-adipocytes. **D.** 3T3-L1 adipocytes were infected as described in C. Viable counts (CFU) were quantified by plating serial dilutions of cell lysates (open bars), and total counts (CEQ) were quantified by qPCR (grey bars). **E.** 3T3-L1 adipocytes were infected as described in C. In order to kill extra-cellular bacteria cells were pre-incubated with 200 µg/ml amikacin 2 h prior lysis and plating. Results are expressed as % of bacterial load at day 0. **F.** 3T3-L1 adipocytes and pre-adipocytes were infected as described in C. Cells were treated with 1 µg/ml rifampicin (Rif), 0.1 µg/ml isoniazid (Inh), 25 µg/ml pyrazinamide (Pza) or 5 µg/ml ethambutol (Emb) at day 3, and bacterial loads were quantified at day 5. Results are expressed as % of bacterial load at day 5 in the absence of antibiotics (Ø). In all panels, results are the mean of three independent experiments and bars indicate ± sd. NT, not tested; NS, not significant, and *, p<0.05, as assessed by Mann-Whitney test of median comparison.

The ability of adipocytes to act as macrophage (Mφ)-like and to phagocytose particles, including live microorganisms, has been demonstrated recently using the 3T3-L1 murine cell line as a model [13], [14]. Under appropriate stimulation, 3T3-L1 cells can differentiate into adipose cells similar to those of the white adipose tissue [15]. In order to determine whether mycobacteria could bind to adipocytes and, if so, which cellular receptor(s) might be involved in this process, we carried out cold binding assays by incubating live *M. tuberculosis* (clinical strain MT103) with 3T3-L1 adipocytes in the presence or absence of various inhibitors. Cells were infected at a multiplicity of infection (MOI) of 1 bacterium per cell. After 4 h at 4°C, nearly 10% of the initial inoculum (9,600 ± 500 colony-forming units, CFUs, per 100,000 cells, noted as 100% on Figure 1A) were found attached to the cells in the absence of inhibitor. As mycobacteria are exceptionally rich in glyco-conjugates, especially in mannosylated compounds that can interact with a variety of host cell surface lectins [16], we considered yeast mannan and *M. tuberculosis* lipoarabinomannan (LAM), a major mannose-rich lipoglycan of the mycobacterial envelope, as possible inhibitors in the assay. None of these compounds could inhibit mycobacterial binding to the cells (Figure 1A), suggesting that mannose residues of the bacterial cell envelope are not involved in the binding process. Adipocytes express numerous scavenger receptors (SRs), and mycobacterial binding to SRs on monocyte-derived Mφs has been reported [17]. We thus next considered SRs as candidate *M. tuberculosis* receptors on adipocytes, and we included SR ligands as possible inhibitors in the assay. Interestingly, the polyanionic SR ligands polyinosinic acid (polyI) and fucoidan, as well as 1-palmitoyl-2-azelaoyl phosphocholine (PAz-PC), one of the predominant oxidized low density lipoprotein (OxLDL) species [18], could inhibit mycobacterial binding to the cells of 25%, 60%, and 90%, respectively (Figure 1A). As a control, polycationic ligands, such as polyadenosinic acid (polyA), had no effect on the binding process (Figure 1A). Similar results were obtained in human primary adipose cells extracted from plastic surgery wastes and infected as described above. Again, SR ligands, such as polyI, fucoidan and PAz-PC could inhibit mycobacterial attachment to adipocytes of up to 80%, while polyA had no effect on the binding process (Figure 1A). These results demonstrate that *M. tuberculosis* can bind to adipocytes of murine or human origin through SRs. Interestingly, the most potent binding inhibitors in adipocytes, namely fucoidan and PAz-PC, had no effect on mycobacterial attachment to 3T3-L1 pre-adipocytes (Figure 1B).

### *M. tuberculosis* persists in a non-replicating state within adipocytes

**Figure 2:**
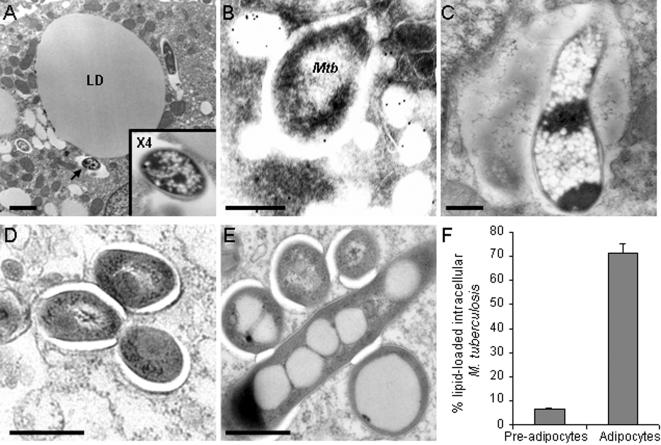
*M. tuberculosis* accumulates lipid droplets inside adipocytes. 3T3-L1 mouse adipocytes **(A–C&E)** and pre-adipocytes **(D)** were pulsed with *M. tuberculosis* MT103 at a MOI of 1 for 4 h, then chased with fresh medium for 24 h and observed by electron microscopy. In A., the arrow indicates a mycobacterial vacuole shown at higher magnification in the inset; LD, lipid droplet; bar = 2 µm. In B., perilipinA/B was immuno-stained with gold-conjugated antibodies. *Mtb*, *M. tuberculosis*; bar = 200 nm. In C, bar = 500 nm. In D&E, bar = 200 nm. **F.** Electron micrographs of 2 independent experiments were used to quantified lipid droplet accumulation inside mycobacteria when within adipocytes and pre-adipocytes. An average of 50 mycobacteria were observed in different fields in each sample.

We next evaluated the ability of adipose cells to phagocytose mycobacteria by pulsing infected 3T3-L1 cells at 37°C. After 24 h infection, observation of the cells under the electron microscope clearly showed intracellular bacilli within membrane-bound vacuoles (Figure 2A–E). In some cases, mycobacterial vacuoles were found to fuse with lipid droplets (Figure 2B), as revealed by immuno-detection of perilipins, that are specific to the surface of lipid droplets in adipocytes [19]. A few bacilli were observed inside vacuoles whose amorphous lumen resembled lipid droplet content, and that most likely resulted from the fusion between the mycobacterial vacuoles and pre-existing lipid droplets (Figure 2C); in these cases, mycobacteria were heavily loaded in small electron-dense vesicles resembling lipid inclusions. Whereas mycobacteria barely carried lipid inclusions in pre-adipocytes (Figure 2D&F), they were commonly loaded in such inclusions in mature adipocytes (Figure 2E&F).

Pathogenic mycobacteria are known for their ability to parasitize Mφs, which are the first *M. tuberculosis* host cells in the lungs, and to multiply within these cells with a replication time of about 20 h [20]. Apart from Mφs, other cell types, including fibroblasts and epithelial cells, have been shown to be permissive to mycobacterial replication [21], [22], [23]. In order to evaluate whether mycobacteria could also replicate in adipocytes, 3T3-L1 adipocytes were pulsed with live mycobacteria for 4 h at 37°C at a MOI of 1, and chased with culture medium. At various time-points after infection, cells were lysed and their bacterial content was measured by plating lysates onto agar and scoring CFUs. Surprisingly, mature adipocytes were not permissive to mycobacterial growth over a 10-day period (Figure 1C). Interestingly, the mycobacterial stasis observed in adipocytes correlated with cell differentiation and accumulation of lipid droplets, as fibroblast-like pre-adipocytes allowed mycobacterial replication with a doubling time of about 40 h (Fig. 1C), which is less than the commonly observed values in macrophages. No obvious cytotoxicity was noticed in pre-adipocytes along infection. In order to investigate whether mycobacteriostasis corresponded to genuine persistence of the bacillus in a non-dividing state, or to a mix of killing and replication, we quantified the number of chromosome equivalents (CEQs) in infected 3T3-L1 adipocytes following a recently described procedure [11]. Our results thus suggest that *M. tuberculosis* persists in a non-replicating state in adipocytes (Figure 1D). Treatment of the infected cells for 2 h with a mycobactericidal dose of the membrane-impermeant antibiotics amikacin prior to cell lysis and bacteria plating indicated that persisting bacilli were mostly intracellular (Figure 1E). We next reasoned that anti-mycobacterial drugs, such as isoniazid (INH), known to act exclusively on non-dormant actively dividing bacilli [24] should have only moderate effects, if any, on intra-adipose cell mycobacteria. On the other hand, drugs such as pyrazinamide (PZA), known to affect cell viability in dormant bacilli [25], should prove efficacious at killing *M. tuberculosis* when inside adipocytes. We infected adipocytes with *M. tuberculosis* as described above, and treated the cells with INH at day 3 after infection, and evaluated the bacterial load of the cells at day 5. INH had no significant effect on *M. tuberculosis* in adipocytes (Figure 1F). By contrast, the bacterial load was reduced by 5–10 fold in 3T3-L1 pre-adipocytes infected and treated in the same manner (Figure 1F). An opposite pattern was observed for PZA, that showed no significant effect on mycobacterial viability in pre-adipocytes, but reduced, significantly although less than rifampicin and ethambutol, the bacterial load in mature adipocytes of 30–50% (Figure 1F). We also assessed the ability of two other major anti-mycobacterial drugs commonly used to treat TB, namely rifampicin (RIF) and ethambutol (EMB), to inhibit mycobacterial persistence in adipocytes. Both EMB and RFP could reduce bacterial load of 80–90% in both adipocytes and pre-adipocytes (Figure 1F).

### *M. tuberculosis* is present in adipose tissue from individuals with latent or active TB

**Figure 3:**
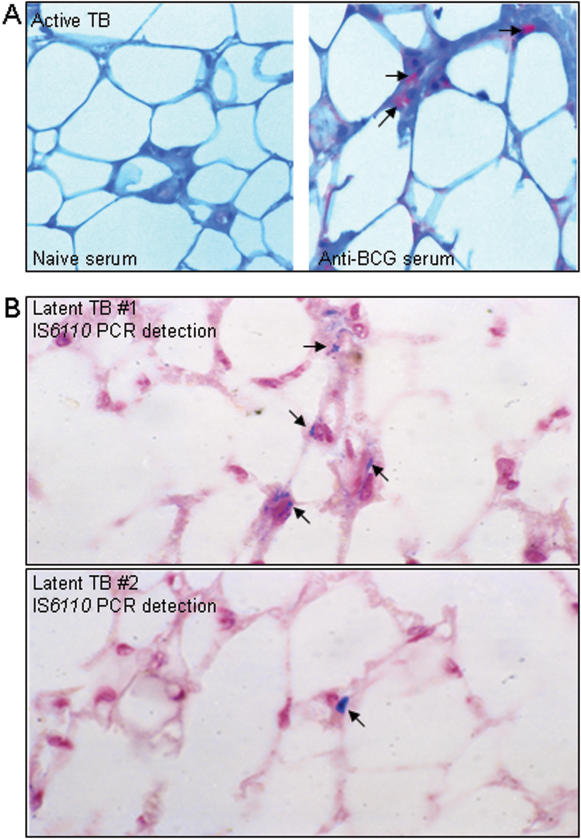
Detection of *M. tuberculosis* in adipose tissue from individuals with latent or active TB. **A.** Perinodal adipose tissue was taken from biopsy of the mediastinal lymph node from a patient with active TB. Bacilli were immuno-detected using an anti-BCG rabbit hyperimmune serum (right panel). As a control, a serial section was stained with serum from a naïve animal (left panel). **B.** Perinodal adipose tissue was taken at autopsy from two individuals (upper and lower panels) with no clinical sign of pulmonary TB. *In situ* PCR was used to detect IS*6110*. In A&B, arrows indicate positive signals.

Finally we wished to evaluate whether adipose tissue could be targeted by *M. tuberculosis* during natural infection in humans. To accomplish this we obtained peri-nodal adipose tissue biopsy samples from 3 patients with active TB. Immuno-detection of *M. tuberculosis* using an anti-BCG hyperimmune serum revealed the presence of the bacillus in samples from one individual (Figure 3A). Although this result, due to the limited number of samples available, does not allow us to make a general statement about infection of adipose tissue during active TB, it does indicate that the bacillus can access this tissue, at least around the mediastinal lymph node during active disease.

In order to evaluate whether *M. tuberculosis* might persist in adipose tissue in individuals with latent TB, we obtained 57 peri-renal, abdominal and peri-nodal adipose tissue samples from 19 autopsy cases of Mexican individuals who died from causes other than TB, and we proceeded to detect mycobacterial DNA (IS*6110*) by *in situ* as well as conventional PCR. Nine samples (representing 6 individuals) showed positivity for mycobacterial DNA by either or both techniques (illustrated in Figure 3, recorded in Table 1). Since these individuals had no sign of TB at autopsy, it is likely that they carried latent infections. However, TB is highly endemic in Mexico and we cannot exclude that those individuals with PCR positivity had not been infected recently before they died. We thus made similar investigations using 26 autopsy samples from 20 French individuals who died in Paris where TB is not endemic. As illustrated in Table 2, we could show, by both *in situ* and conventional PCR, the presence of *M. tuberculosis* DNA in 4 samples (representing 4 individuals) taken from heart, kidney, skin and lymph node adipose tissue. A total of 8 samples (representing 6 individuals) were found positive by either or both technique. Some discrepancy between the two techniques have already been reported [8], and are likely due to the different autopsy sample slices used to realize the assays. Although DNA detection does not formally prove the presence of the bacillus in a viable form, as compared to culture or inoculation to guinea pigs, it suggests past exposure.

**Table 1:**
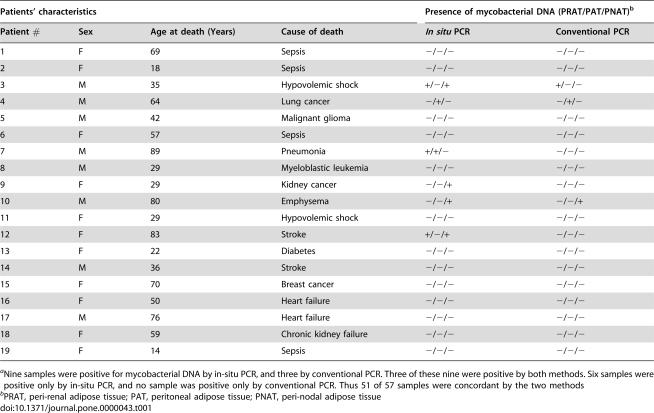
Detection of *M. tuberculosis* DNA in adipose tissue from Mexican autopsy samples^a^

Patients' characteristics				Presence of mycobacterial DNA (PRAT/PAT/PNAT)^b^	
Patient #	Sex	Age at death (Years)	Cause of death	*In situ* PCR	Conventional PCR
1	F	69	Sepsis	−/−/−	−/−/−
2	F	18	Sepsis	−/−/−	−/−/−
3	M	35	Hypovolemic shock	+/−/+	+/−/−
4	M	64	Lung cancer	−/+/−	−/+/−
5	M	42	Malignant glioma	−/−/−	−/−/−
6	F	57	Sepsis	−/−/−	−/−/−
7	M	89	Pneumonia	+/+/−	−/−/−
8	M	29	Myeloblastic leukemia	−/−/−	−/−/−
9	F	29	Kidney cancer	−/−/+	−/−/−
10	M	80	Emphysema	−/−/+	−/−/+
11	F	29	Hypovolemic shock	−/−/−	−/−/−
12	F	83	Stroke	+/−/+	−/−/−
13	F	22	Diabetes	−/−/−	−/−/−
14	M	36	Stroke	−/−/−	−/−/−
15	F	70	Breast cancer	−/−/−	−/−/−
16	F	50	Heart failure	−/−/−	−/−/−
17	M	76	Heart failure	−/−/−	−/−/−
18	F	59	Chronic kidney failure	−/−/−	−/−/−
19	F	14	Sepsis	−/−/−	−/−/−

a Nine samples were positive for mycobacterial DNA by in-situ PCR, and three by conventional PCR. Three of these nine were positive by both methods. Six samples were positive only by in-situ PCR, and no sample was positive only by conventional PCR. Thus 51 of 57 samples were concordant by the two methods

b PRAT, peri-renal adipose tissue; PAT, peritoneal adipose tissue; PNAT, peri-nodal adipose tissue

**Table 2:**
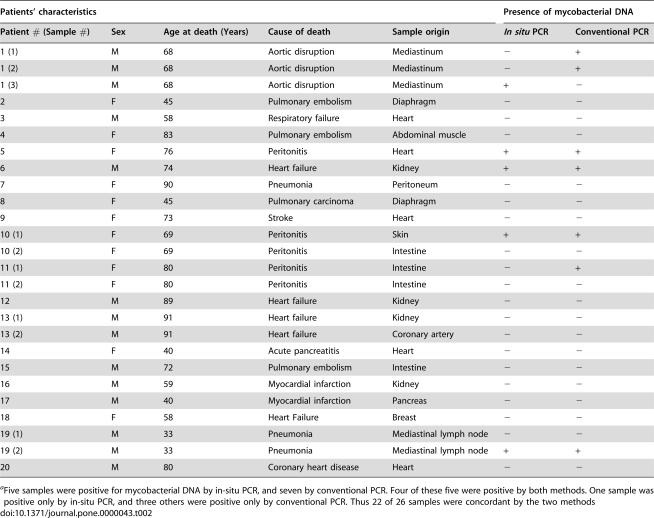
Detection of *M. tuberculosis* DNA in adipose tissue from French autopsy samples^a^

Patients' characteristics					Presence of mycobacterial DNA	
Patient # (Sample #)	Sex	Age at death (Years)	Cause of death	Sample origin	*In situ* PCR	Conventional PCR
1 (1)	M	68	Aortic disruption	Mediastinum	−	+
1 (2)	M	68	Aortic disruption	Mediastinum	−	+
1 (3)	M	68	Aortic disruption	Mediastinum	+	−
2	F	45	Pulmonary embolism	Diaphragm	−	−
3	M	58	Respiratory failure	Heart	−	−
4	F	83	Pulmonary embolism	Abdominal muscle	−	−
5	F	76	Peritonitis	Heart	+	+
6	M	74	Heart failure	Kidney	+	+
7	F	90	Pneumonia	Peritoneum	−	−
8	F	45	Pulmonary carcinoma	Diaphragm	−	−
9	F	73	Stroke	Heart	−	−
10 (1)	F	69	Peritonitis	Skin	+	+
10 (2)	F	69	Peritonitis	Intestine	−	−
11 (1)	F	80	Peritonitis	Intestine	−	+
11 (2)	F	80	Peritonitis	Intestine	−	−
12	M	89	Heart failure	Kidney	−	−
13 (1)	M	91	Heart failure	Kidney	−	−
13 (2)	M	91	Heart failure	Coronary artery	−	−
14	F	40	Acute pancreatitis	Heart	−	−
15	M	72	Pulmonary embolism	Intestine	−	−
16	M	59	Myocardial infarction	Kidney	−	−
17	M	40	Myocardial infarction	Pancreas	−	−
18	F	58	Heart Failure	Breast	−	−
19 (1)	M	33	Pneumonia	Mediastinal lymph node	−	−
19 (2)	M	33	Pneumonia	Mediastinal lymph node	+	+
20	M	80	Coronary heart disease	Heart	−	−

a Five samples were positive for mycobacterial DNA by in-situ PCR, and seven by conventional PCR. Four of these five were positive by both methods. One sample was positive only by in-situ PCR, and three others were positive only by conventional PCR. Thus 22 of 26 samples were concordant by the two methods

## Discussion

In summary, this study reveals that *M. tuberculosis* can persist without replication inside adipocytes *in vitro* and likely in adipose tissue *in vivo* in naturally infected humans with either active or latent TB. Our *in vitro* results are reminiscent of those of two previous studies reporting that neither the vaccine strain *Mycobacterium bovis* bacille Calmette-Guérin (BCG) nor *Mycobacterium leprae*, the etiological agent of Hansen's disease or leprosy, could multiply when cultivated onto adipose tissue, whereas the bacteria replicated well in a variety of other tissues [26], [27]. Here, we have shown that *M. tuberculosis* enters into adipocytes after binding to SRs. The finding that PAz-PC, one of the major species of OxLDL inhibits *M. tuberculosis* binding to adipose cells may suggest participation of CD36 in the binding process, as this particular SR has been reported to mediate internalization of OxLDL by adipocytes [28], [29]. After entry, we have shown that mycobacteria can persist within adipocytes without apparent replication. This was not the case in pre-adipocytes in which mycobacteria could multiply, which might rely on the different receptors used by the bacillus to enter the two cell types.

This phenomenon could also depend on the host cell lipid metabolism as only mature adipocytes, but not fibroblastic pre-adipocytes, could restrict mycobacterial growth. Inside adipocytes, mycobacteria were found to accumulate electron-dense lipid droplets. Recently, it has been suggested that sequestration of host fatty acids in cytoplasmic lipid droplets by mycobacteria may allow the bacilli to survive in a non-replicating state [30]. More generally, accumulation of triacylglycerols inside the cytoplasm of various actinomycetes is thought to be associated with bacterial survival under conditions of stress, such as limited aeration [31]. Our results are also reminiscent of a previous study in which the ability of two calixarenes, namely HOC-12.5 (macrocyclon) and HOC-60, to respectively inhibit and enhance *M. tuberculosis* growth in Mφs was correlated to the ability of these drugs to respectively induce and prevent accumulation of lipid droplets within the cells [32]. Altogether, the previous studies and our results stress the importance of the host cell lipid metabolism in mycobacterial infections. In adipose cells, it is likely that mycobacteria use lipolytic enzymes to degrade host cell triglycerides and import fatty acids in their cytoplasm. Analysis of the *M. tuberculosis* complete genome has revealed that the bacillus possesses nearly 20 putative lipases [33]. The use of *M. tuberculosis* lipase-deficient mutants will be useful to delineate the metabolic interactions between the bacillus and adipose cells.

Mycobacterial behaviour within adipocytes may be interesting to further investigate as a model of persistence, as compared to other currently used *in vitro* models of mycobacterial dormancy. Adipocyte differentiation is associated with high O_2_ consumption and hypoxia is known to prevent triglyceride accumulation in differentiating adipose cells and adipogenesis [34]. An attractive hypothesis is that access of mycobacteria to O_2_ may be limited within adipocytes, which may induce mycobacterial dormancy. In this view, it will be interesting to compare *M. tuberculosis* metabolism in adipose cells to that of dormant bacilli in the O_2_ deprivation-induced Wayne's dormancy model [24].

The adipose tissue is distributed throughout the body and represents 15–25% of the total body mass. This tissue may thus constitute, among others, a vast mycobacterial reservoir, allowing the bacilli to persist virtually anywhere in the body. In this aspect, it is interesting that we could show the presence of *M. tuberculosis* DNA in adipose tissue samples of various anatomical origins taken at autopsy in a public hospital in Paris. Since TB is not endemic in Paris, it is unlikely that those individuals with PCR positivity had been infected recently before they died. In particular, they showed no sign of pulmonary TB at autopsy. Although DNA detection does not formally prove the presence of the bacillus as compared to culture or inoculation to guinea pigs, it suggests past exposure. In an attempt to detect intact bacilli in these PCR-positive samples, we performed Ziehl-Nielsen staining. Unfortunately no sample could be stained using this technique (data not shown). This is likely due to the low bacterial load within the samples, and/or to modification of the cell wall of dormant bacilli [35], [36], [37].

Reactivation of persistent *M. tuberculosis* infection in the adipose tissue might account, at least in part, for extra-pulmonary TB, which represent about 15% of the total reactivation cases [9], [10]. How, following entry by the respiratory route, the bacilli subsequently reach extra-pulmonary adipose tissue remains to be determined. However, we can anticipate that both hematogenous and cell-mediated (*eg* by migrating dendritic cells) routes contribute to this process. Once inside adipocytes, we have shown that the tubercle bacillus exhibit increased and diminished resistance to INH and PZA, respectively, which might consistent with a dormancy-like state of the bacilli inside the cells. However, another possible explanation might rely on the differential hydrophilicity of these antibiotics. RFP and EMB are lipophilic, while INH is hydrophilic. This will have to be further explored, for instance by examining the levels of antibiotics inside the cells. It is also known that PZA has a better antimicrobial activity at acidic pH. Whether *M. tuberculosis* resides in an acidified intracellular compartment inside mature adipocytes remains to be studied. If genuine, these findings might have to be taken into account for the future design of antibiotics-based therapeutic strategies. For instance, the use of INH only in preventive therapy, especially in HIV-infected individuals, might not be sufficient to prevent reactivation of latent infection of the adipose tissue. Investigating these aspects will require the development an appropriate animal model of intra-adipose tissue mycobacterial persistence in order to study resistance to antibiotics, efficacy of various types of immune responses, and reactivation factors. Mycobacterial persistence within cells or tissue with limited antigen presentation abilities ([38] and data not shown) may be of great advantage for the tubercle bacillus. This may prove challenging for the design of future vaccines to combat persistent mycobacteria and prevent reactivating TB. Finally, our results may show relevance for other mycobacterial diseases, likely affecting the adipose tissue, such as Hansen's disease and Buruli ulcer.
